# Leptomeningeal Metastases in a Patient with an Extragonadal Germ Cell Tumor

**DOI:** 10.4137/cmo.s687

**Published:** 2008-04-24

**Authors:** Yoshiaki Kinebuchi, Masakuni Ishikawa, Osamu Ishizuka, Osamu Nishizawa, Kazuhiro Hongo

**Affiliations:** 1 Department of Urology, Shinshu University School of Medicine, Matsumoto, Japan; 2 Department of Neurosurgery, Shinshu University School of Medicine, Matsumoto, Japan

**Keywords:** leptomeningeal metastasis, brain metastasis, germ cell tumor, surgery, chemotherapy

## Abstract

We present a case of leptomeningeal metastases in a 30-year-old man with an extragonadal germ cell tumor. The patient was referred to our hospital for treatment of an occipital brain metastasis. This lesion was resected, followed by whole brain radiotherapy and further chemotherapy, and a temporary complete remission was achieved. However, leptomeningeal recurrence developed, and despite salvage chemotherapy, the patient died of disease. Although multidisciplinary treatment is given to treat brain metastases of germ cell tumors, the patients’ prognosis has been unsatisfactory. The identification of a standard/effective treatment is required.

## Introduction

Extragonadal germ cell tumors (GCTs) belong to poor prognosis, according to the International Germ Cell Consensus Classification (IGCCC), and the presence of brain metastasis is predictive to worse prognosis. Recently, multidisciplinary approaches involving combination chemotherapy, irradiation, and surgery have been used to treat patients with brain metastasis, and successful cases have been reported (Bokemeyer et al. 1997; Fossa et al. 1999; Mahalati et al. 1999; Lutterbach et al. 2002). However, the long-term survival of such patients is not yet satisfactory. Intrathecal metastasis (meningeal dissemination) of GCT is rare, and no effective treatment has been established. We present a case that temporarily responded to chemotherapy and discuss the possible treatment options in such cases.

## Case Report

**Figure 1:**
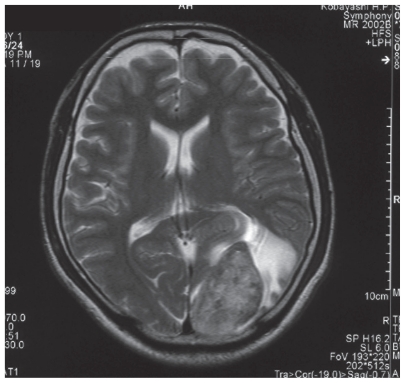
Brain MRI. The solitary metastatic lesion was shown, which was 4.5 cm × 3 cm in diameter, occupying the left occipital lobe (before operation).

**Figure 2:**
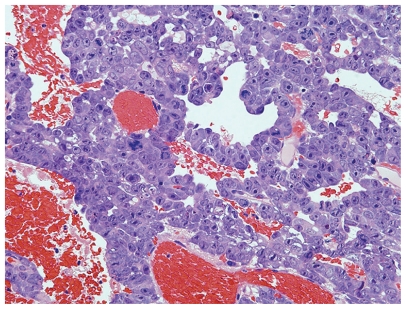
H&E staining showed solid and tubular tumor cells with large, bizarre nuclei, coexisted with hemorrhage (magnification, × 100). Immunohistochemical staining showed tumor cells to be positive with CD30 and AFP that suggested the embryonal carcinoma with yolk sac tumor component, and partially positive with HCG, suggesting syncytiotrophoblastic cells.

A 30-year-old man with an extragonadal GCT of mediastinal origin complained of right homonymous hemianopia and was referred to Shinshu University Hospital for the treatment of the brain metastasis. About 4 months earlier, the patient had received induction chemotherapy with three courses of BEP (bleomycin, etoposide, and cisplatin) for multiple lung metastases in another hospital. The brain lesion had grown rapidly (Fig. 1), and serum tumor markers were elevated, including lactate dehydrogenase (LDH) at 447 U/l (normal, 118–236), αfetoprotein (AFP) at 480 ng/ml (normal ≦10), and human chorionic gonadotropin (HCG) at 629 IU/l (normal ≦1.0). The patient had immediate neurosurgery to remove the tumor. On pathology, the tumor was diagnosed as metastatic embryonal carcinoma and yolk sac tumor (Fig. 2). The patient also received whole brain radiation therapy (WBRT) (total 30 Gy/10 fractions) and adjuvant chemotherapy consisting of two courses of VIP therapy (etoposide, ifosfamide, and cisplatin). All tumor markers normalized. The residual lung tumors were resected, and no tumor remnant was seen on pathology.

**Figure 3:**
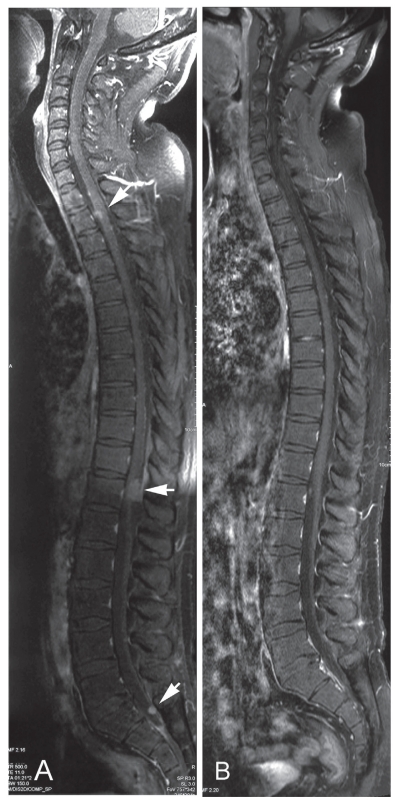
MRI of the spinal cord before and after salvage chemotherapy. **3A**-Well-enhanced, multiple leptomeningeal metastases were detected at the 2nd and 12th thoracic cord levels, as well as at the 1st sacral cord level (Th2, Th12, and S1) (white arrows). **3B**-Complete remission of the leptomeningeal metastases was demonstrated after salvage chemotherapy.

One month after lung surgery, the patient developed lumbago and a gait disturbance. On magnetic resonance imaging (MRI), multiple leptomeningeal metastases with no brain recurrence were seen (Fig. 3A). Salvage chemotherapy, consisting of combination irinotecan and nedaplatin therapy, was given. After four courses of therapy, the leptomeningeal lesions disappeared (Fig. 3B). However, the serum HCG elevated again, and recurrent cerebral ventricular and meningeal lesions appeared within a short period of time. Despite the use of additional salvage chemotherapy involving three courses of TIN therapy (paclitaxel, ifosfamide, and nedaplatin), in combination with the peripheral blood stem cell transplantation, the patient died due to disease progression 12 months after the initial brain surgery.

## Discussion

Brain metastasis from malignant GCT occurs in 1%–3% of patients (Bokemeyer et al. 1997; Fossa et al. 1999). Multidisciplinary treatment, involving chemotherapy, irradiation, and surgery, is required to treat brain GCT metastases. The prognosis of patients with brain metastasis is poor, but it is relatively better in patients with a solitary (isolated) metastasis and in those with an initial metastasis compared to patients with multiple lesions or a relapse (Bokemeyer et al. 1997; Fossa et al. 1999; Mahalati et al. 1999; Lutterbach et al. 2002). Fossa et al. reported that the 5-year survival rate of patients with an initial metastasis was 45%, but that of patients with recurrence after induction chemotherapy was 12% (Fossa et al. 1999). According to guidelines of the American National Cancer Institute (NCI) and the European Urological Association, the standard treatment for GCT brain metastasis is chemotherapy in conjunction with WBRT (NCI, 2008; Albers et al. 2005). On the other hand, Salvati et al. recommended that, if the brain tumor is resectable, aggressive surgical treatment followed by WBRT and/or adjuvant chemotherapy should be given (Salvati et al. 2006).

The incidence of leptomeningeal metastasis of primary central nervous system (CNS) tumors has been reported to range from 7% to 27%, but extra-CNS GCT metastasis is rare (Engelhard et al. 2005). There has been only one report in the last 10 years (Miranda et al. 2005). The etiology of leptomeningeal metastasis has been considered to due to cerebrospinal fluid (CSF) dissemination by direct contact with the tumor or by surgical inoculation (Engelhard et al. 2005). The treatment of leptomeningeal metastasis is difficult. Intrathecal injection of anti-cancer drugs such as methotrexate has been reported, but the effectiveness of this treatment in GCT is not proven (Engelhard et al. 2005). Peereboom examined the brain capillary permeability of anti-cancer agents and reported that etoposide, cisplatin, irinotecan, and bleomycin had intermediate permeability, and that the combination of these drugs might have some effect (Peereboom, 2005). Jahnke et al. reported the efficacy of intraarterial chemotherapy in conjunction with transient osmotic blood brain barrier disruption for primary CNS germ cell tumors (Jahnke et al. 2007). However, long-term survivors without progression had been observed only in patients with pure germinoma. Recently, the efficacy of new anti-cancer agents, such as paclitaxel, gemcitabin, oxaliplatin, and irinotecan, as well as the combination of these drugs, has been reported for cisplatin refractory disease, and their use is expected to improve the prognosis (Kollmannsberger et al. 2006). Various combinations have been used, including irinotecan with nedaplatin (or cisplatin), TIN, or TIP (paclitaxel, ifosfamide, and cisplatin) (Miki et al. 2002; Nonomura et al. 2007).

In the present case, these regimens were applied as salvage chemotherapy, and nedaplatin was selected instead of cisplatin, that had been used in previous therapies. Although the tumor initially responded well, recurrence occurred within a short period of time, and it became difficult to continue chemotherapy because of adverse effects and impairment of the patient’s performance status. Brain surgery was initially done due to rapid tumor growth and the risk of brain hemorrhage. The role of adjuvant WBRT after surgery remains to be clarified. In general, conventional fractionation (viz. 2 Gy/fractions to ≥40 Gy) is usually recommended for brain metastasis in order to decrease late neurotoxicity (Soffietti et al. 2006). Despite the use of postoperative adjuvant therapy, intrathecal metastases developed. Craniospinal radiotherapy should be identified as another treatment option for meningeal metastases. However, it might be difficult to deliver in the context of myelo-suppression from heavy pre-treatment with chemotherapy in the present case. If another chemotherapy regimen including new anti-cancer drugs had been used as second-line therapy, or if a greater number of chemotherapy courses had been given after the brain surgery, the result might have been different.

Further clinical studies and the establishment of a management strategy for CNS metastasis in GCT patients are required.
